# Usher Syndrome: Genetics of a Human Ciliopathy

**DOI:** 10.3390/ijms22136723

**Published:** 2021-06-23

**Authors:** Carla Fuster-García, Belén García-Bohórquez, Ana Rodríguez-Muñoz, Elena Aller, Teresa Jaijo, José M. Millán, Gema García-García

**Affiliations:** 1 Molecular, Cellular and Genomics Biomedicine Research Group, Instituto de Investigación Sanitaria La Fe (IIS La Fe), 46026 Valencia, Spain; c.fustergarcia@gmail.com (C.F.-G.); belen_garcia@iislafe.es (B.G.-B.); rodriguezmunoz.ana@gmail.com (A.R.-M.); aller_ele@gva.es (E.A.); jaijo_ter@gva.es (T.J.); gegarcia@ciberer.es (G.G.-G.); 2 Unidad Mixta de Enfermedades Raras IIS La Fe-Centro de Investigación Príncipe Felipe, 46026 Valencia, Spain; 3 Biomedical Research Network for Rare Diseases, Hospital Universitario y Politécnico La Fe, 46026 Valencia, Spain; 4 Genetics Unit, Hospital Universitario y Politécnico La Fe, 46026 Valencia, Spain

**Keywords:** deafblindness, inherited retinal dystrophy, retinitis pigmentosa, sensorineural hearing loss, inner ear, photoreceptor, variant curation, pathogenic variant

## Abstract

Usher syndrome (USH) is an autosomal recessive syndromic ciliopathy characterized by sensorineural hearing loss, retinitis pigmentosa and, sometimes, vestibular dysfunction. There are three clinical types depending on the severity and age of onset of the symptoms; in addition, ten genes are reported to be causative of USH, and six more related to the disease. These genes encode proteins of a diverse nature, which interact and form a dynamic protein network called the “Usher interactome”. In the organ of Corti, the USH proteins are essential for the correct development and maintenance of the structure and cohesion of the stereocilia. In the retina, the USH protein network is principally located in the periciliary region of the photoreceptors, and plays an important role in the maintenance of the periciliary structure and the trafficking of molecules between the inner and the outer segments of photoreceptors. Even though some genes are clearly involved in the syndrome, others are controversial. Moreover, expression of some USH genes has been detected in other tissues, which could explain their involvement in additional mild comorbidities. In this paper, we review the genetics of Usher syndrome and the spectrum of mutations in USH genes. The aim is to identify possible mutation associations with the disease and provide an updated genotype–phenotype correlation.

## 1. Introduction 

Usher syndrome (USH) is the most common cause of genetic deafblindness and follows an autosomal recessive inheritance. This disorder is characterized by the combination of a degenerative vision loss condition known as retinitis pigmentosa (RP), sensorineural hearing loss (SNHL) and, sometimes, vestibular dysfunction. USH is considered a rare disease, because it has a low prevalence, which ranges from 3 to 6.2 per 100,000 people [1]. Even though a recent report has cast doubts about the vestibular phenotypic differences among the USH subtypes [2], this disease is traditionally classified into three different subtypes depending on the age of onset, severity and progression of the symptoms, and the presence or absence of vestibular dysfunction [3]. USH type 1 (USH1) is the most severe subtype and is characterized by a severe to profound prelingual SNHL, early RP onset and vestibular alterations. USH type 2 (USH2) is the most frequent subtype and presents with moderate to severe SNHL, onset of RP in the second decade of life, and normal vestibular function. USH type 3 (USH3) is the rarest form and is characterized by postlingual progressive SNHL, and both a variable onset age of RP and vestibular function. Some patients do not fit into these three subtypes and are classified as atypical USH. Due to the increased use of high-throughput sequencing (HTS) as the preferred molecular diagnosis tool for USH, and for other retinal dystrophies and hearing loss (HL) disorders, new reports have been published regarding candidate genes, revised genotype–phenotype correlations and insights into the phenotypic spectrum of the disease. In this review, we gather past knowledge and new genetic and clinical findings to provide an updated picture of the current state of USH.

## 2. The Genetic Heterogeneity of USH

In addition to being clinically heterogeneous, USH is also genetically heterogeneous. To date, 10 genes have been associated with USH (Table 1), and these are typically responsible for one of its three subtypes. *MYO7A*, *USH1C*, *CDH23*, *PCDH15*, *USH1G* and *CIB2* cause USH1; *USH2A*, *ADGRV1* and *WHRN* are accountable for USH2; and *CLRN1* is the only gene associated with USH3 thus far. Moreover, some of these genes are also involved in other non-syndromic pathologies. Mutations in *USH1C*, *CDH23*, *PCDH15*, *USH1G*, *CIB2*, *WHRN*, *PDZD7* and *MYO7A* can cause non-syndromic recessive HL [4,5,6,7,8,9,10,11,12], and the latter is also associated with a dominant form of HL [13]. In addition, *USH2A* and *CLRN1* are also related to non-syndromic RP [14,15].

As mentioned above, USH can be caused by mutations in at least ten genes, and each of these is usually accountable for one specific category of the disease. However, the correlation between gene and phenotype is not entirely rigid. The recent increase in the number of genetically screened cases has revealed that some patients harbor mutations in USH genes usually associated with a different subtype. For example, variants in *MYO7A* and *CDH23* have been identified in patients diagnosed with USH2 [16,17,18,19,20], and USH1 and USH3 cases have been found to be caused by mutations in *USH2A* [16,17,20].

## 3. The USH Genes

### 3.1. MYO7A

*MYO7A*, consisting of 49 exons and encompassing an 87 kb genomic region, was the first gene to be associated with USH [21,22]. *MYO7A* is the most prevalent gene among USH1 cases—pathogenic variants in this gene comprise 50% of the cases included in this subtype [23]—and it is also the second most frequently mutated gene of all USH cases [24]. Moreover, it has been reported that mutations in *MYO7A* can also cause autosomal dominant and recessive HL and atypical USH [25,26,27]. *MYO7A* encodes the myosin VIIA protein [21,22], which is included in the family of the called unconventional myosins, motor proteins that carry out intracellular molecular transport by linking to actin. The major isoform consists of 2215 amino acids [28] and the encoded protein has a motor head domain that interacts with actin, followed by a protein–protein interaction domain and ending in a tail responsible for binding to other cellular proteins [29,30,31,32]. Myosin VIIA is expressed in the photoreceptors of the retina, the RPE cells, and the inner ear [33,34,35]. In the retina, this protein is responsible for transporting opsin from the inner to the outer segments [36,37], whereas in the cochlea its function consists of linking the stereocilia tip links, resulting in the maintenance of the hair bundle unity [38,39].

### 3.2. USH1C

The genomic region of the *USH1C* gene extends to 51 kb and contains a total of 28 exons, eight of which having alternative splicing [40,41]. This gene has been described as causative of USH1 and non-syndromic HL [42]. Mutations in coding regions that are alternatively spliced (exons 15 and 22 to 28) have been related to non-syndromic HL [43] and mutations in constitutive coding to USH1. However, several studies have also suggested that variants in the alternatively spliced exons could be responsible for atypical USH cases [44,45]. The *USH1C* gene encodes for the harmonin protein, consisting of 899 amino acids, and a variety of scaffold proteins, all of which contain PDZ domains that are in charge of organizing protein complexes, and binding with USH1 and USH2 proteins in both the inner ear and retina [41,46,47].

### 3.3. CDH23

*CDH23* is the second most frequently mutated gene in USH1 cases and has also been associated with non-syndromic autosomal recessive HL [48]. Missense mutations that allow a residual protein function to be maintained that is sufficient for the correct retina function have been predominantly related to HL, whereas truncating variants are responsible for USH1 [49,50,51,52]. This gene encompasses a 300 kb genomic sequence that has a coding region of 69 exons and two microexons of solely 6 and 3 bp. Three different isoforms are encoded by *CDH23*: isoform a contains 3354 amino acids and is the longest, with a total of 27 extracellular Ca^2+^-binding domains, which differentiates it from isoform b, that has only seven, and c, that has none. Both a and b isoforms are in charge of binding the stereocilia through a tip link in unison with protocadherin 15 in the inner ear. They also play a role in shaping the outer segment as a result of its expression in the inner and outer segment, and in the calyceal processes [53,54,55]. Contrary to the latter two isoforms, and due to the absence of extracellular Ca^2+^-binding domains, isoform c is a cytoplasmic protein that manages the microtubule network settlement [56].

### 3.4. PCDH15

The *PCDH15* gene, which comprises a genomic region of 980 kb formed by 33 exons, is associated with both USH1 and non-syndromic HL, similar to *CDH23* [57,58,59]. Despite its large genome sequence, the intronic sequence can be up to 150 kb, which results in the longest isoform of 1955 amino acids. *PCDH15* belongs to the cadherin superfamily and encodes the protocadherin protein, which, by alternative splicing, results in several isoforms with different functions [57,60]. In both inner ear and photoreceptors, protocadherin 15 shares almost all of its functions with cadherin 23. It has been suggested that it is implicated in the elasticity in mechanotransduction in the tip links in addition to the conservation of calyceal pathways of photoreceptors [55,61,62,63,64,65].

### 3.5. USH1G

*USH1G* is the smallest USH gene, having a 7 kb genomic region and containing three exons, two of which are coding. It has been associated with USH1 and atypical USH, but, in recent years, it has also been described as possibly being responsible for non-syndromic HL [66,67,68]. SANS is a protein of 461 amino acids encoded by *USH1G* and, as a scaffold protein, it contains SAM domains and ankyrin repeats. This protein takes part in tip link maintenance and acts as a vehicle for molecules across the microtubules [69,70,71,72,73,74].

### 3.6. CIB2

The *CIB2* gene comprises only six exons and it was last of the USH-labelled genes because of the findings of a consanguineous family. Several years later, this gene was also associated with non-syndromic HL [75,76,77,78]. Contrary to HL, the implication of *CIB2* in USH cases has been questioned, including in the study of Riazuddin et al., suggesting that the USH family with *CIB2* mutations may be a disease phenocopy. However, the implication of *CIB2* in the inner ear and retina seems to be correct [79]. As its name indicates, *CIB2* codifies the calcium and integrin binding protein 2, which consists of EF hand Ca^2+^-binding domains.

### 3.7. USH2A

*USH2A* is the most common mutated gene among USH cases and was the first gene described as being responsible for USH2 [80,81], which is highly related to the prevalence of the c.2299delG change [82,83]. Moreover, *USH2A* is also implicated in non-syndromic RP cases [84,85]. As one of the largest USH genes, it encompasses a genomic sequence of 800 kb and a coding region of 72 exons. The first 21 exons give rise to the extracellular isoform a with 1546 amino acids [86,87]. The longer isoform (isoform b) includes additional 51 exons and produces a transmembrane protein with a length of 5202 amino acids [88]. In addition, an alternative splicing of exon 71 results in an isoform whose expression is specific to the inner ear [89]. In the inner ear, isoform b cooperates with the ADGRV1 protein to join the stereocilia near its emergent region [89,90,91]. In the retina, its localization in the enveloping region between the connecting cilium and the inner segment of photoreceptors is related to the transport of the molecules to the outer segment and the preservation of these cells in time [92]. Contrarily, isoform a is limited to basement membranes in both stereocilia and photoreceptors [93].

### 3.8. ADGRV1

The *ADGRV1* gene, previously known as *VLGR1* and *GPR98*, is the second most prevalent gene in USH2 cases and was first described in 2004 [94]. This gene is considered to have the largest coding sequence among the USH genes [95,96]. *ADGRV1* encodes the adhesion G-protein coupled receptor V1, whose largest isoform includes 90 exons (6306 amino acids), although it codifies two more isoforms. Growth and organization of stereocilia is carried out by this protein in cooperation with usherin. Adhesion G-protein coupled receptor V1 cooperates in nearly all of the functions of usherin due to their same localization [71,90,97].

### 3.9. WHRN

The *WHRN* gene is responsible for USH2 cases [98]. Its coding sequence is alternatively spliced, producing several isoforms, among which two, known both as whirlin, are the most significant [27]. The 12 exons included in the coding sequence of *WHRN* are translated into the longest isoform, which has a length of 907 amino acids, whereas the shortest begins in the sixth exon, and thus lacks two PDZ domains of the *N*-terminal region [99]. It has been observed that mutations affecting the common PDZ domain in both isoforms produce non-syndromic HL, whereas those localized to the remaining protein domains cause USH2 [100,101,102,103,104,105]. This is supported by findings in mice that displayed alterations in the auditory system when the short isoform was altered, and because of the unique expression of the longest isoform in the retina, which demonstrates that mutations in the two final PDZ domains cause USH2 [106,107]. Whirlin is involved in diverse functions not only in the inner ear and photoreceptors, but also in brain development [106]. Whirlin anchors usherin and ADGRV1 proteins by their cytoplasmic regions in the inner ear; the shortest whirlin mediates the regulation of stereocilia growth via its expression in the tip links, and the largest manages the hair bundle organization [108,109,110,111]. In addition, in the retina, whirlin expresses in the connecting cilium and also localizes to the synaptic region [112]. 

### 3.10. CLRN1

*CLRN1* is the only gene known to be causative of USH3 and has also been described as being responsible for nonsyndromic RP. Even though mutations in *CLRN1* are rare, the prevalence of this USH subtype reaches around 40% in the Finnish population [113,114]. Its sequence of approximately 18 kb contains four exons and codes for clarin1, a protein with four transmembrane domains [115]. The 11 different splice variants described could explain the wide clinical spectrum reported in USH3 patients [116]. Among the different speculations about its function, it has been concluded that clarin1 expresses in the synapse areas in both cochlea and photoreceptors [117,118,119].

### 3.11. Other Related Genes

Other genes have been related to USH, but their association with the disease remains unclear due to some contradictions between the symptoms and the few existing reports. A homozygous variant in *ABHD12*, a gene more commonly associated with PHARC syndrome and RP, was identified in one family that was clinically diagnosed with USH3 [120]. However, these patients underwent clinical examination after the genetic findings, in which one of the two affected members was additionally diagnosed with ataxia. This was consistent with a variant of PHARC and led the authors to propose the neurological syndrome should be taken into account as a differential diagnosis for USH3 cases. The *HARS* gene was also proposed to be causative of USH3 based on three patients from two different Amish communities carrying the same missense mutation [121]. Nevertheless, no further USH cases due to mutations in *HARS* have been reported, and other studies have related the gene to other neuropathies [122,123,124,125]. It is thus unclear whether *HARS* is actually an USH gene or involved in a wider phenotypic spectrum. *CEP250* was first associated with atypical USH, supported by a phenotype characterized by early onset HL and relatively mild RP [126]; however, two later studies with findings related to the same gene revealed that the ophthalmological features of the respective patients corresponded to a cone-rod dystrophy rather than RP [20,127]. The affected members of the index family presented by Khateb and colleagues were also carriers of a *PCARE* pathogenic mutation, which could have masked the actual pure *CEP250* phenotype or had an epistatic effect. Consequently, *CEP250* is now categorized as an USH-like gene. Recently, three studies with comparable cases identified *ARSG* mutations in atypical USH patients, designated as USH type 4 [128,129,130]. This appears to be a robust new candidate gene responsible for the disease, and *ARSG* should be included in upcoming genetic screenings. Even though *CEP78* was labelled as being causative of atypical USH in a study by Fu and colleagues, the clinical features of the supporting cases of this research and of two previous studies were consistent with cone-rod dystrophy and SNHL [131,132,133]. Thus, similar to the case of *CEP250*, *CEP78* is another gene responsible for an USH-like phenotype rather than actual USH. Finally, *ESPN* has been associated with different forms of HL and with one case matching USH1 [134]. Furthermore, an interaction of ESPN with WHRN has been reported [135]. Thus, it remains to be seen if this gene will also have a wider disease spectrum and be attributable to USH. In addition to these genes, it should also be noted that *PDZD7*, further to its association with HL, was proposed as an *USH2A* modifier and, with *ADGRV1*, a possible participant in digenic inheritance [136].

## 4. The USH Interactome

**Figure 1:**
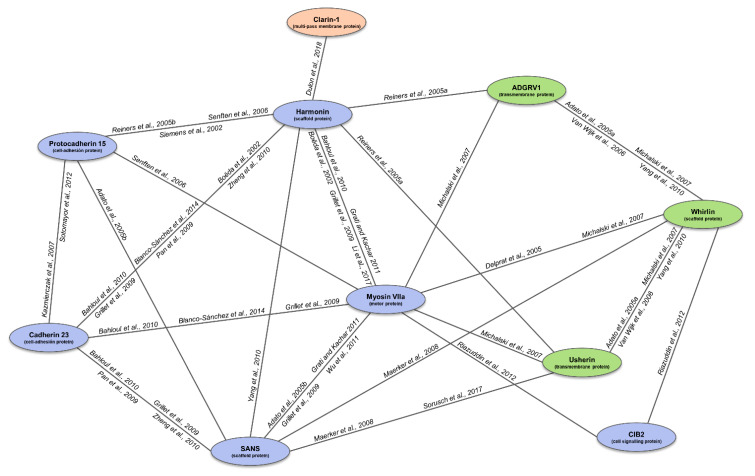
Representation of the USH interactome and supporting literature. The connecting lines represent the detected interactions between the USH1 (blue), USH2 (green) and USH3 (orange) proteins, according to the in vitro and in vivo studies to date (specified next to the lines). Adapted figure from Fuster García (2020). References figure: [10,92,106,107,108,138,139,140,141,142] [25,26,28,29,143,144,145] [61,146,147,148].

USH proteins interact directly with at least one other, thus building a protein network called the “USH interactome” (Figure 1) [137]. This protein complex can be explained because the alteration of any of the proteins results in the manifestation of almost the same clinical signs. It has been reported in several studies that this protein network is mainly present in the stereocilia of hair cells and the periciliary region of photoreceptors. In 2014, it was reported that the interactome was also associated with other proteins related to ciliopathies and retinopathies [73]. The USH interactome core is formed by whirlin, harmonin, and SANS. In the inner ear, the extracellular unions produced by cadherin 23 and protocadherin 15 are linked in this scaffolding core by the tethering with the cytoskeleton or other proteins, such as myosin VIIA. In addition, the interactome is also located in the synaptic region and the periciliary membrane of photoreceptors [1,73].

## 5. Is USH Actually a Ciliopathy?

The cells affected by USH are the photoreceptors in the retina, and the hair cells of the cochlea and vestibular system, each of which presents ciliary-like structures, namely, the stereocilia and the connecting cilium, respectively. Regarding the inner ear, technically only the vestibular sensory cells present a genuine microtubule-based cilium at the vertex of the V-shaped hair bundle, which is the kinocilium; this bundle is only present during development in the cochlear hair cells, before subsequently disappearing [149,150]. Nonetheless, this cilium is present during certain stages, and the stereocilia-microvilli resemble ciliary formations. Consequently, many specialists consider USH to be a ciliopathy, which is defined as a group of pathologies that arise from defects in genes that participate in the structure or signaling of cilia [73]. Nonetheless, the use of the term ciliopathy to define USH is controversial. Even though some authors do not include USH in this category [151,152,153], others incorporate USH into the retinal ciliopathies group [154,155,156]. Even though the USH proteins are not ciliary proteins, such as those encoded by the genes *RPGR*, *RPGRIP1* or *CEP290*, or the Bardet Biedl Syndrome genes (*BBS* family genes), they are clearly essential for the function of photoreceptor cilia and USH should at least be considered a second-order ciliopathy [152].

Numerous tissues have cilia; thus, any genetic variation that alters ciliary functions could affect different organs. Several additional signs have been described in USH patients related to cilia function: bronchiectasis [157], olfactory loss [158,159], subclinical nasal ciliary beat frequency [160], nociception [161], reduced sperm motility [162] and somatosensory deficit [163,164,165]. However, further evidence is still necessary to consider these characteristics as being USH-related comorbidities.

## 6. Animal Models

Animal models have contributed significantly to elucidate the molecular mechanisms of Usher syndrome and have been widely used in the development of therapeutic strategies to preserve both vision and hearing (Table 2) [166].

The first USH animal models manifested features and conducts corresponding to a hearing loss phenotype, such as deafness, circling behavior and head-tossing movements. Due to the high conservation between the genetic pathways that regulate auditory perception in mice and humans, all current available USH mutant mice well mimic the impairment of human hearing. Each of these reproduces the characteristic early-onset hearing loss and vestibular defects as found in USH1 patients; however, none of these mouse models shows retinal degeneration, with the exception of the Ush1c knock-in mouse, generated to study the c.216G>A mutation, which displays hearing loss, vestibular defects and retinal degeneration [167]. A spontaneous mutant USH2 mouse model (kunming) showed early-onset retinal degeneration, but harbored mutations in two genes implicated in inherited retinal dystrophies: Ush2a and Pde6b [168]. In addition, a Ush2a knock-out mouse presented only mild late-onset retinal degeneration [93]. The third model is a murine model that has a disruption of the *N*-terminal PDZ domains of whirlin, which recapitulates the human USH2 phenotype [106].

In general, USH mouse models poorly recapitulate the visual deficiencies manifested in human patients when the orthologous genes are disrupted. This could be due to the fact that, in mouse photoreceptors, the calyceal processes and periciliary membranes are absent or underdeveloped compared to human photoreceptors [54,169]. However, these structures are present and well developed in frogs, fishes, chicks and primates [170].

The second most commonly used animal as a model of USH is the zebrafish. Even though the evolutionary distance of zebrafish from human may pose problems for the study of the disease, most developed models showed early-onset retinal degeneration, which, as previously mentioned, is lacking in most mouse models [171]. The zebrafish ush2a knock-out also recapitulated the auditory abnormalities and later onset of retinal degeneration characteristic of USH2 patients [172].

Despite the numerous USH models described to date, new animal and cellular models are necessary to increase our understanding of the cellular and molecular processes implicated in vision and hearing, and to enable better diagnosis and treatment of patients with USH.

## 7. Mutational Spectra in the USH Genes: Types, Distribution and Genotype-Phenotype Correlations

Given the increase in new molecular findings relating to USH, one aim of this study is to review the current gene associations with the disease and to provide an updated genotype–phenotype correlation, including the functional implication of the known pathogenic variants at the transcriptomic and proteomic levels, to better understand the syndromic spectrum and to improve the disease diagnosis. For this analysis, we focus only on the *MYO7A*, *CDH23*, *ADGRV1*, *USH1C*, *USH2A* and *PCDH15* genes, excluding the remaining four due to their limited number of mutations, which prevents a robust statistical evaluation. 

**Figure 2:**
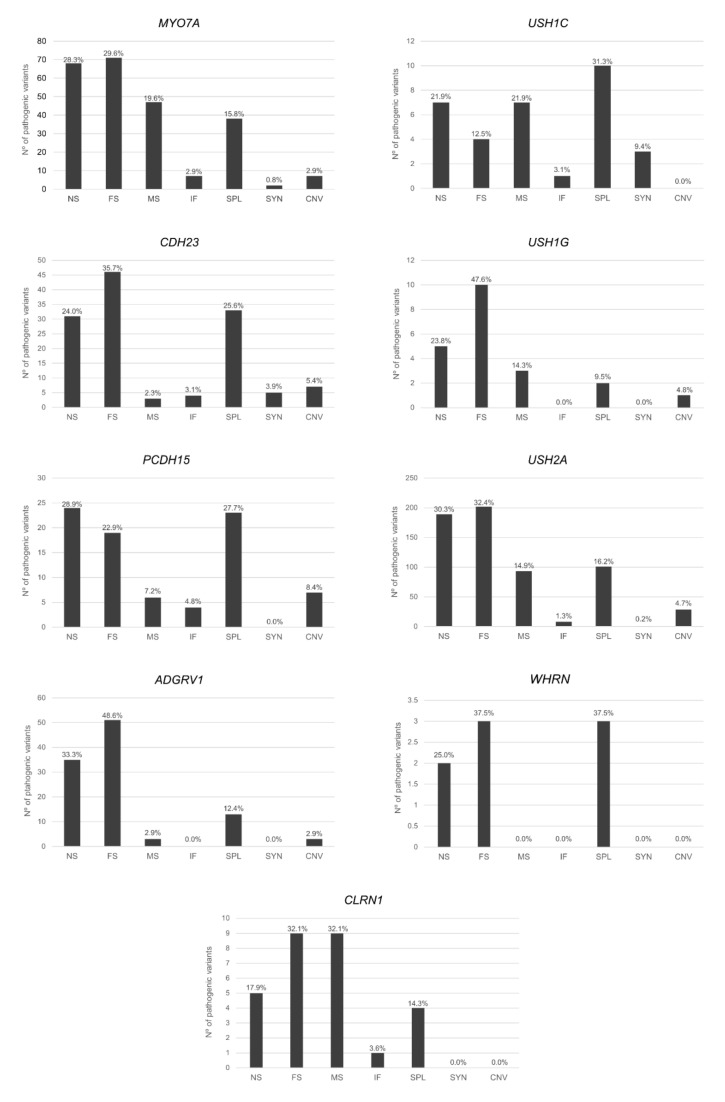
Mutation count in USH genes (*MYO7A*, *USH1C*, *CDH23*, *PCDH15*, *USH2A*, *ADGRV1*). Abbreviations: NS, nonsense variant; FS, frameshift variant; MS, missense variant; IF, in-frame indel variant; SYN, synonymous; SPL, splicing variant.

**Figure 3:**
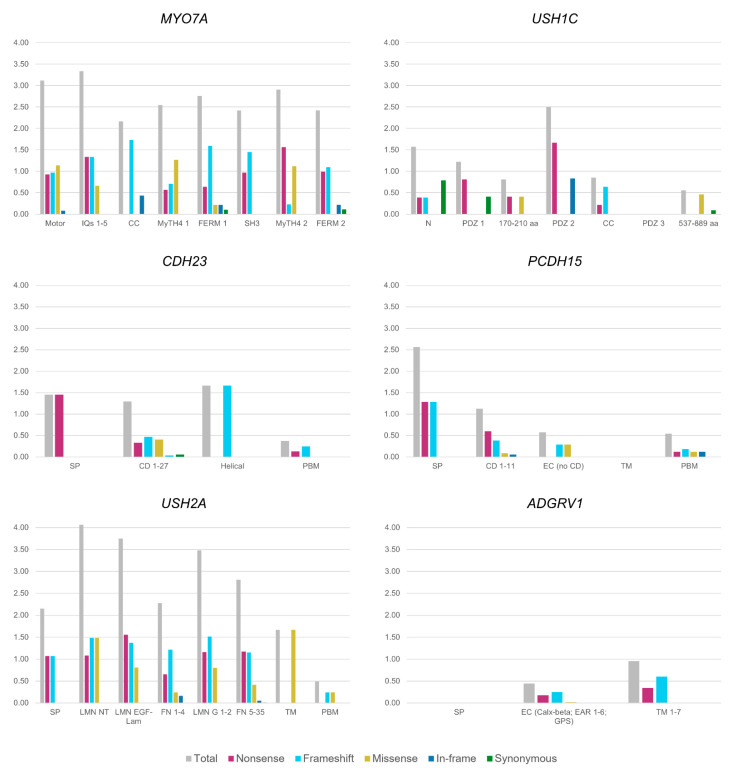
Mutational ratio in USH protein domains. Mutational ratio for the protein domains for the genes *MYO7A*, *USH1C*, *CDH23*, *PCDH15*, *USH2A* and *ADGRV1*, shown on the y-axis. Ratios are calculated counting the number of pathogenic variants of each type located in each domain and normalized with respect to the size of the specific domain. Abbreviations: Motor, motor domain; IQ, IQ calmodulin-binding motif; CC, coil-coiled domain; MyTH4; Myosin Tail Homology 4 domain; FERM, FERM domain (F for 4.1 protein, E for ezrin, R for radixin and M for moesin); SH3; Src Homology 3 domain; N, *N*-terminal domain; PDZ, PDZ domain; aa, amino acids; SP, signal peptide; PBM, PDZ binding domain; CD, cadherin domains; EC, extracellular domain; TM, transmembrane; LMN NT, laminin *N*-terminal domain; LMN EGF-Lam, laminin EGF-like domain; LMN G; laminin G domain; FN, fibronectin domain.

**Figure 4:**
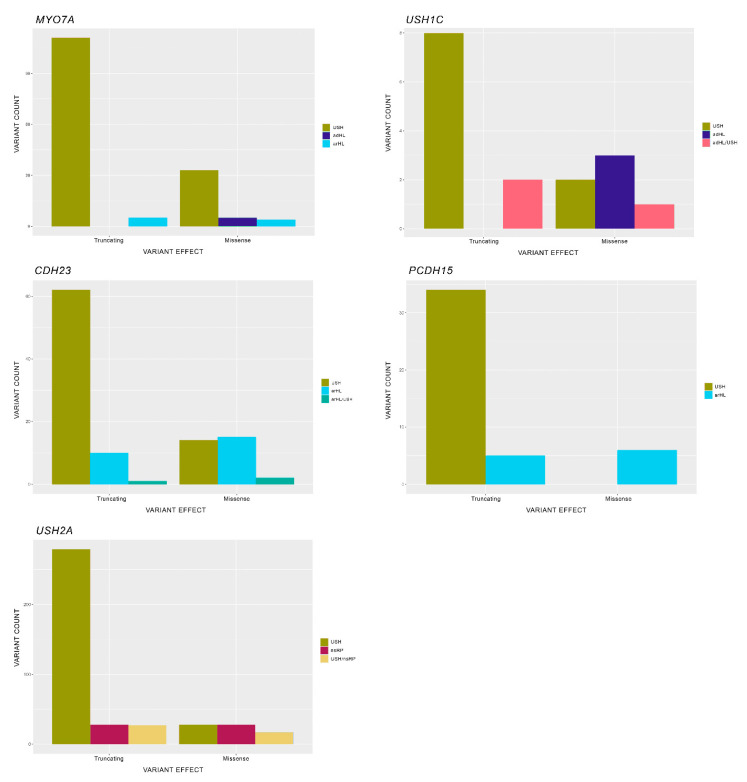
Mutational spectrum per gene for each phenotype. Abbreviations: USH, Usher syndrome; arHL, autosomal recessive hearing loss; adHL, autosomal dominant hearing loss; nsRP, non-syndromic retinitis pigmentosa. *ADGRV1* is not included in the representation because all mutations obtained from the database were responsible for only USH and not for additional phenotypes.

Our study is based on a specific Usher Syndrome database (https://grenada.lumc.nl/LOVD2/Usher_montpellier/USHbases.html, accessed on 6 July 2020; version of July 2020), which collects both genotypic and phenotypic up-to-date information about the USH genes; results are shown in Figure 2, Figure 3 and Figure 4. 

### 7.1. Type of Mutations

It is well known that the USH genes have a significant mutational heterogeneity because pathogenic variants of different natures have been identified, in which the pertinent protein is altered by different molecular pathways. Moreover, in recent years, five deep-intronic variants in *USH2A* and one in *CLRN1* have been identified and functional analyses have demonstrated their implication in the splicing process by the inclusion of a pseudoexon (PE) in the mRNA, leading to a truncating effect [46,203,204,205]. More deep-intronic mutations are expected to be found in the future, further adding to the genetic heterogeneity.

The heterogeneous distribution of every type of mutation described to date in each gene is depicted in Figure 2, which provides an overall representation of nonsense, frameshift, and splicing variants. Furthermore, the large size of the intronic regions in some genes related to a high number of repetitive regions, such as *PCDH15* and *USH2A*, has led to more cases of large rearrangements compared to the other genes [206,207,208,209]. As a result, CNVs should be heeded in the capture designs and analyses by HTS or by implementing the method with supplementary techniques, such as Multiplex Ligation-dependent Probe Amplification or Comparative Genomic Hybridization arrays.

### 7.2. Mutation Distribution among the USH Proteins Domains

In addition to the mutation heterogeneity itself, the distribution of mutations throughout the protein sequence and, therefore, the effect on a specific domain, should also be taken into account to explain the functional impact of each type of mutation. 

We were interested in studying the possible associations between two categorical variables: domain vs. type of mutation. For each gene, we counted the number of pathogenic variants of each type located in each domain. To take into account the size of the domains, the number of mutations of each type was normalized to the size of the specific domain. To simplify the analysis and data representation, consecutive domains belonging to the same typology were grouped. A Pearson’s chi-squared goodness-of-fit test (based on the chi-squared statistical test) was performed with the aim of calculating the difference among expected and observed values for each type of mutation in the different gene domains. The aim of a goodness-of-fit test is to estimate whether the theoretical or expected distribution is a good representation of the real data distribution; that is, to assess if the observed data are consistent with either a theoretical or an expected distribution.

#### 7.2.1. USH2A

The results revealed significant differences for missense variants whose proportion is higher in the laminin *N*-terminal and the laminin G-like domains (*p*-value: 0.001147) (Figure 3). In an allelic study of *USH2A* carried out by Lenassi et al., the missense variants present in their cohort were mostly located in laminin G-like domains, as shown in this review, and in fibronectin type III domains. In our analysis, fibronectin type III domains presented a significantly lower proportion of missense mutations, which may have been a consequence of grouping all fibronectin type III domains to more easily represent the data (Figure 3).

In contrast, some domains accumulate a significantly smaller number of missense variants than expected. The signal peptide only accumulates nonsense and frameshift mutations (Figure 3), suggesting that the significant difference observed for missense frequency (*p*-value: 0.001147) may be related to the possible tolerance for this type of variant in this domain.

Notably, in *USH2A* the different localization of a certain type of mutation determines not only its pathogenicity, but also the clinical phenotype depending on the photoreceptor function that is altered. Yu and colleagues, in 2020, proposed two possibilities by which some mutations are associated with RP and USH; the first is related to the two usherin isoforms, and the second to the *C*- and *N*-terminal interactions in the retina and inner ear [210]. Nevertheless, more studies are required to understand the underlying reason why different pathologies can be caused not only by mutations with the same localization, but also by the same missense variant.

#### 7.2.2. PCDH15

No significant difference existed among each type of mutation distribution and each protein domain in *PCDH15*. However, we found a high proportion of nonsense variants in CD domains, and a low frequency of nonsense variants in extracellular and cytoplasmic domains, which was close to being significantly different from expectations (*p*-value: 0.0516) (Figure 3).

#### 7.2.3. MYO7A

In *MYO7A*, as observed in *USH2A*, significant differences exist for missense variants, with a higher proportion in the motor domain and the MyTH4-1 and MyTH4-2 domains (*p*-value: 0.002281) (Figure 3).

In contrast, it must be noted that in the SH3 domain only truncating mutations have been described and the frequency of missense variants is significantly lower than the expected number (*p*-value: 0.002281) (Figure 3). The number of missense variants was also lower than expected for Coiled Coil and FERM domains.

#### 7.2.4. ADGRV1 

In cytoplasmatic domains 2–4 (included in the transmembrane region in Figure 3), the observed frequency of nonsense variants was higher than expected (*p*-value: 0.002952). In the McMillan et al. study conducted in 2004 [211], the generated mouse model that encoded only the ectodomains of the *ADGRV1* gene manifested audiogenic seizures in a manner that clarified the implication of cytoplasmic and transmembrane domains in the auditory function [212]. 

#### 7.2.5. USH1C

The statistical analysis showed significant differences in the number of nonsense variants: the frequency was higher than expected in the PDZ1 and PDZ2 domains (*p*-value: 0.015) (Figure 3). Protein interactions occur through unions among the PDZ domains, which are essential to protein complex arrangements [42], meaning that null variants located in these regions truncate the protein and, therefore, these interactions. 

#### 7.2.6. CDH23

Any type of mutation showed significant differences with respect to their localization in the different domains. Notably, in the signal peptide and the helical region of *CDH23*, only truncating mutations have been identified (Figure 3). However, only one mutation for each region has been described to date, and we could not identify any significant differences.

### 7.3. Types of Mutations and Their Influence in the Phenotype

Notably, establishing correlations in recessive pathologies between genotype and phenotype is complicated when a major proportion of the study group comprises compound heterozygotes. This is particularly the case if most alleles are private to each family, as in USH. 

To investigate whether there is an association between the type of mutation depending on their protein effect and the phenotype, a Pearson’s chi-squared test was applied using these two categorical variables. Due to the low number of some types of variants, different variants were grouped into two categories depending on the effect at the protein level: missense and truncating. A second analysis was carried out by combining the type of mutation with the domain in which it was located, to compare it with the phenotype it resulted in; however, we did not obtain any significant results.

#### 7.3.1. USH2A

Pearson’s chi-squared test showed a relationship between both considered variables for the *USH2A* gene (*p*-value = 1.604 × 10^−15^. There was a higher observed frequency of missense mutations in the nsRP phenotype than expected and a lower missense variant percentage in the USH phenotype than expected; both of these differences were significant. Moreover, the frequency of truncating variants in the nsRP phenotype was significantly fewer than expected (Figure 4 and Figure S1). These findings are in agreement with those observed in other studies for *USH2A*.

In 2015, Lenassi et al. established an allele hierarchy of *USH2A* variants in 457 patients from three different cohorts [15], to identify the mutations that resulted in either RP or USH. Their results showed that, although null alleles were responsible for the syndromic form, missense variants were associated with RP in a higher proportion compared to USH, and the phenotype depended on the second causal variant. As mentioned previously, our results also indicate a high prevalence of truncating variants in USH cases in contrast to the higher number of missense variants in RP cases (Figure 4). 

Similarly, a cohort of 57 patients carrying p.Cys759Phe (p.C759F) was studied using HTS by Pérez-Carro et al. with the purpose of clarifying the role of this variant as either a RP-causing variant or a possible phenotype modifier [212]. In this study, patients were classified into three categories: the first included patients homozygous for the p.C759F variant; the second contained compound heterozygous patients for p.C759F with another missense variant; and the third compiled compound heterozygous patients for p.C759F with a truncating mutation. Their findings showed that carrying a truncating variant was sufficient to develop an USH phenotype, whereas the other two categories of combinations resulted in the milder form of non-syndromic RP or RP with a late onset hypoacusis. 

#### 7.3.2. PCDH15

In *PCDH15*, the analysis showed significant differences between the considered categories (*p*-value = 2.604 × 10^−5^. The observed frequency of missense variants was higher than expected in the DFNB phenotype and lower in the USH phenotype (Figure 4 and Figure S1).

Previous studies have also described an association between missense mutations and non-syndromic deafness in PCDH15 [60,213].

#### 7.3.3. MYO7A 

The analysis showed a significant higher proportion of missense variant for the DFNA phenotype than expected (*p*-value: 0.0002928) (Figure 4 and Figure S1). A similar association between missense and HL has been previously reported [5,49,214].

In contrast, a recent study of 53 patients with biallelic *MYO7A* mutations did not find any statistically significant difference between phenotypic features and the type of mutation [215]. 

#### 7.3.4. CDH23

As in the case of *MYO7A*, statistical analysis showed significant differences, and indicated a higher frequency of missense variant for the DFNB phenotype in *CDH23* than expected (*p*-value: 0.0001546) (Figure 4 and Figure S1).

In a similar study aiming to determine the phenotype–genotype correlation and a mutation-dependent prognosis for *CDH23*, Schultz and colleagues found that heterozygous genotypes consisting of an autosomal recessive HL allele in trans with an USH allele preserved visual and vestibular functions; hence, for this gene it appears that alleles associated with HL are phenotypically dominant over those causatives of the syndromic form. Even though all of the HL alleles were missense variants (except one amino acid in-frame deletion), USH alleles were of any type, but dominated by those alleles that led to a truncated protein [52].

#### 7.3.5. USH1C

Pearson’s chi-squared test determined that there was an association between the type of mutations and the different phenotypes (*p*-value: 0.04224). However, Pearson’s residual analysis could not determine more specific associations (Figure 4 and Figure S1). 

## 8. Conclusions

As expected, mutation heterogeneity remains a challenge to understanding how the protein and mutation nature correlate with the phenotype, particularly when the complete genotype is not accessible. Establishing a genotype–phenotype correlation is a challenging task for inherited retinal dystrophies. The high number of genes involved, and the large size of some of these genes, have hampered the mutational screening of large cohorts of patients, thus hindering the determination of these statistical relationships. The advent of HTS has allowed massive screenings that, together with a well-curated database such as LOVD, circumvents this problem. 

In addition, the rapidly evolving field of gene therapy highlights the importance of an exhaustive molecular diagnosis, because these diagnoses can be directed toward a specific gene or are even mutation dependent. The most promising strategies as potential treatments for USH include approaches based on gene augmentation, antisense oligonucleotides (AONs) and gene editing using the CRISPR/Cas system [166]. Several clinical trials are currently underway using some of these strategies, for USH and other retinal diseases. Among these, a form of gene therapy via gene supplementation (LUXTURNA™) has received FDA approval for Leber Congenital Amaurosis with mutations in *RPE65* [216]. This therapeutic strategy, although using dual vectors, is also being used in a European clinical trial for the *MYO7A* gene (UshTher, Horizon, 2020; #NCT02065011). Additionally, other studies are focused on the use of AONs to treat mutations in several USH genes [217,218,219], including a phase I/II clinical trial of patients with mutations in exon 13 of *USH2A* (ProQR, Stellar; #NCT03780257). 

These outstanding scientific advances and the development of innovative therapeutic strategies are leading towards precision medicine. It is thus essential to continue investigating the mechanisms underlying the pathology of USH, performing comprehensive genetic diagnoses and establishing genotype–phenotype correlations that will be indispensable to develop and determine the efficacy of future treatments.

## Figures and Tables

**Table 1:** Summary of the USH genes.

USH Type	Gene	MIM Number	Protein	GRCh38 Coordinates	Function
USH1	*MYO7A*	276900 ^1^ 276903 ^2^	myosin VIIA	chr11:77128246–77215241	actin-based motor protein
	*USH1C*	276904 ^1^ 605242 ^2^	harmonin	chr11:17493895–17544416	scaffold protein
	*CDH23*	601067 ^1^ 605516 ^2^	cadherin 23	chr10:71396934–71815947	cell adhesion
	*PCDH15*	602083 ^1^ 605514 ^2^	protocadherin 15	chr10:53802771–55627942	cell adhesion
	*USH1G*	606943 ^1^ 607696 ^2^	SANS	chr17:74916083–74923256	scaffold protein
	*CIB2*	614869 ^1^ 605564 ^2^	CIB2	chr15:78104606–78131544	calcium and integrin binding protein
USH2	*USH2A*	276901 ^1^ 608400 ^2^	usherin	chr1:215622891–216423448	cell adhesion
	*ADGRV1*	605472 ^1^ 602851 ^2^	adhesion G protein-coupled receptor V1	chr5:90529344–91164437	adhesion G protein-coupled receptor
	*WHRN*	611383 ^1^ 607928 ^2^	whirlin	chr9:114402080–114505473	scaffold protein
USH3	*CLRN1*	276902 ^1^ 606397 ^2^	clarin 1	chr3:150926163–150972999	transmembrane protein

^1^ Phenotype MIM number. ^2^ Gene/Locus MIM number.

**Table 2:** Described animal models of Usher syndrome.

Gene	Animal	Model	HL	VD	RD	References
**USH type 1**						
*MYO7A*	*Danio rerio*	*myo7a*^*m*/*m*^ (mariner)	yes	yes	yes	[173,174]
	*Mus musculus*	*Myo7a*^*sh1*/*sh1*^ (shaker1)	yes	yes	no	[175,176]
		*Myo7a*^*hdb*/*hdb*^ (headbanger)	yes	yes	NA	[177]
		*Myo7a*^*pk*/*pk*^ (polka)	yes	yes	no	[178,179]
		*Myo7a*^*I487N*/*I487N*^ (ewaso)	yes	yes	NA	[180]
		*Myo7a*^*F947I*/*F947I*^ (dumbo)	yes	no	NA	[180]
		*Myo7a* ^−/−^	yes	yes	no	[181]
*USH1C*	*Danio rerio*	*ush1c* ^*fh293*/*fh293*^	yes	yes	yes	[182]
		*ush1c* knock-down	yes	yes	yes	[182]
	*Mus musculus*	*Ush1c*^*dfcr*/*dfcr*^ (deaf circler)	yes	yes	no	[183]
		*Ush1c*^*dfcr*/*dfcr*^ (deaf circler)	yes	yes	no	[183]
		*Ush1c*^*dfcr-2J*/*dfcr-2J*^ (deaf circler 2 Jackson)	yes	yes	no	[183]
		*Ush1c* ^−/−^	yes	yes	no	[184,185]
		*Ush1c* knock-in [c.216G>A]	yes	yes	yes	[167,186]
		*Ush1c*^−/−^*C57Bl*/*6 J*	NA	NA	no	[187]
		*Ush1c*^−/−^*BALB*/*cJ*	NA	NA	yes	[187]
*CDH23*	*Danio rerio*	*cdh23*^*s*/*s*^ (sputnik)	yes	yes	no	[188,189]
	*Xenopus tropicalis*	*cdh23* knock-down	NA	NA	yes	[55]
	*Mus musculus*	*Cdh23*^*v*/*v*^ (waltzer)	yes	yes	no	[190]
*PCDH15*	*Danio rerio*	*pcdh15* ^*p*/*p*^	yes	yes	yes	[191]
	*Xenopus tropicalis*	*pcdh15* knock-down	yes	yes	yes	[55]
	*Mus musculus*	*Pcdh15*^*av*/*av*^ (ames waltzer)	yes	yes	no	[58]
		*Pcdh15*^*I108N*/*I108N*^ (noddy)	yes	yes	NA	[192]
*USH1G*	*Mus musculus*	*Sans*^*js*/*js*^ (jackson shaker)	yes	yes	no	[193]
		*Ush1g*^−/−^*C57Bl*/*6 J*	NA	NA	no	[187]
		*Ush1g*^−/−^*BALB*/*cJ*	NA	NA	yes	[187]
*CIB2*	*Drosophila melanogaster*	CG9236 knock-down	NA	NA	yes	[10]
	*Danio rerio*	*cib2* knock-down	yes	yes	NA	[10]
	*Mus musculus*	*Cib2* ^−/−^	yes	no	no	[77,78]
		*Cib2* ^*tm1a*/*tm1a*^	yes	no	NA	[76]
		*Cib2* knock-in [p.F91S]	yes	no	NA	[76]
**USH type 2**						
*USH2A*	*Danio rerio*	*ush2a* knock-down	NA	NA	yes	[136]
		*ush2a* ^−/−^	yes	yes	yes	[172]
		*ush2a* ^*rmc1*/*rmc1*^	NA	NA	yes	[169]
		*ush2a* ^*b1245*/*b1245*^	NA	NA	yes	[169]
	*Mus musculus*	*Ush2a* ^−/−^	yes	no	yes	[93]
		*KM*^*ush*/*ush*^ (kunming)	yes	no	yes	[168]
*ADGRV1*	*Danio rerio*	*adgrv1* knock-down	NA	NA	yes	[136]
	*Mus musculus*	*Vlgr1* ^*del7TM*/*del7TM*^	yes	no	no	[170]
		*Vlgr1* ^−/−^	yes	no	no	[92,97]
*WHRN*	*Mus musculus*	*Whrn*^*wi*/*wi*^ (whirler)	yes	yes	no	[194,195,196,197]
		*Whrn* ^*L*−/*L*−^	yes	no	yes	[106]
**USH type 3**						
*CLRN1*	*Danio rerio*	*clrn1* knock-down	yes	yes	NA	[117]
		*clrn1* ^−/−^	yes	yes	NA	[198]
	*Mus musculus*	*Clrn1* ^−/−^	yes	yes	no	[118,199]
		*Clrn1* ^*N48K*/*N48K*^	yes	no	NA	[200]
		*Clrn1* ^*ex4*−/−^	yes	NA	NA	[142]
		*Clrn1*^*ex4fl*/*fl*^*Myo15*-*Cre*^+/−^	yes	NA	NA	[142]
		*Clrn1*^−/−^ [KO-TgAC1]	yes	NA	NA	[201]
		*Clrn1* knock-in [N-HA]	no	NA	no	[202]

Abbreviations: HL, hearing loss; VD, vestibular dysfunction; RD, retinal degeneration; NA, not available.

## Data Availability

Not applicable.

## Supplementary Materials

The following are available online at https://www.mdpi.com/article/10.3390/ijms22136723/s1.
