# Supplementary material for: Usher Syndrome: Genetics of a Human Ciliopathy
Source: Int J Mol Sci. 2021 Jun 23;22(13):6723. doi: 10.3390/ijms22136723 (PMC8268283; doi:10.3390/ijms22136723)
Supplement: Supplementary file 1 [file ijms-22-06723-s001.zip › ijms-1252510-supplementary.pdf]

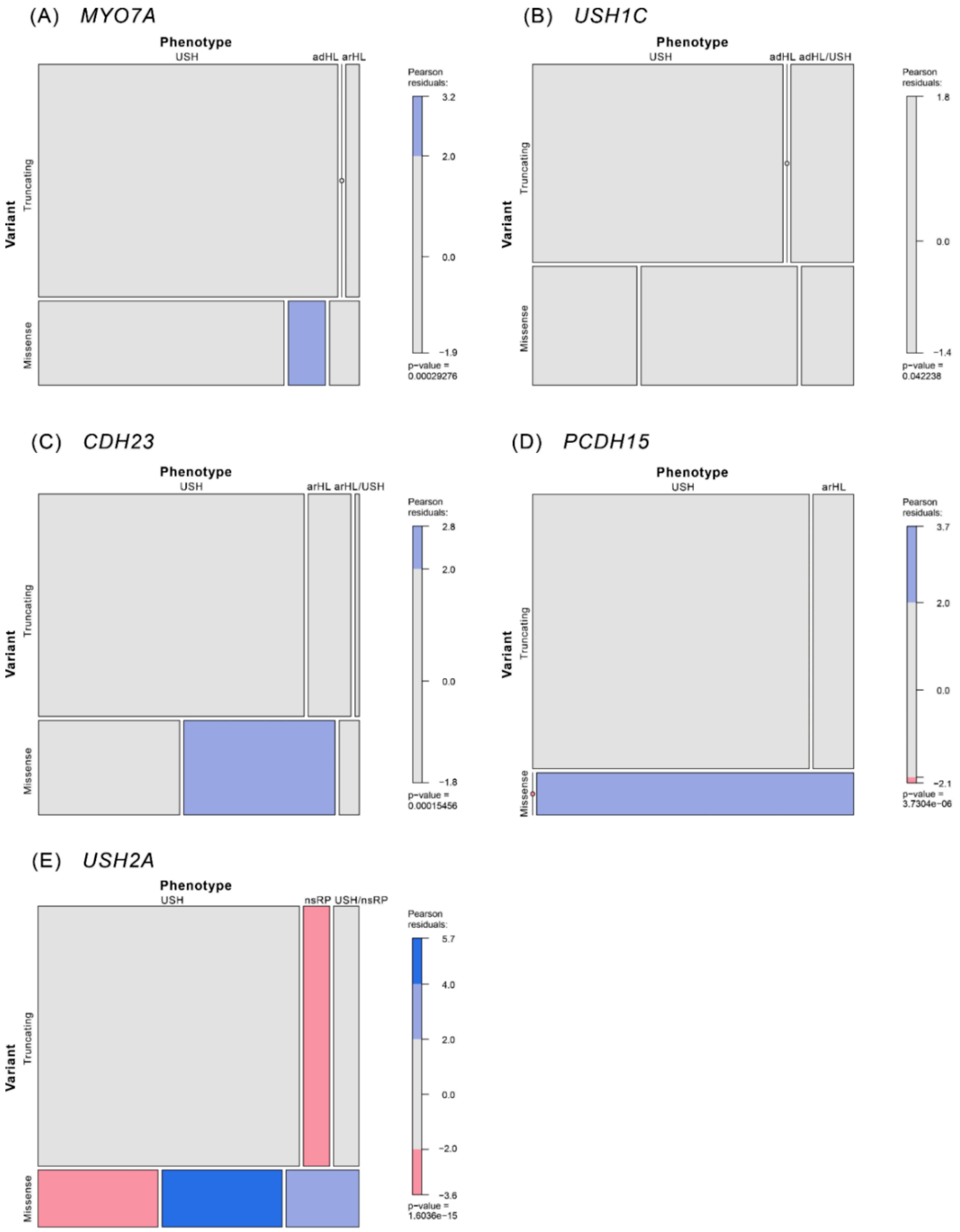

Figure S1. Mosaic representation of Pearson residuals in each gene**.** Pearson's $\chi 2$ test compares the observed frequencies with the expected frequencies. When the test is significant, we continued with the study of the Pearson residuals (which are the difference between the observed and the expected, divided by the square root of the expected). For an α of 0.05, if the residual value exceeds 1.95, it is considered significant. This information is represented by the mosaic graphics.
